# Co-producing a safe mobility and falls informatics platform to drive meaningful quality improvement in the hospital setting: a mixed-methods protocol for the insightFall study

**DOI:** 10.1136/bmjopen-2023-082053

**Published:** 2025-02-03

**Authors:** Rachael Lear, Phoebe Averill, Catalina Carenzo, Rachel Tao, Ben Glampson, Clare Leon-Villapalos, Robert Latchford, Erik Mayer

**Affiliations:** 1 Robin Hood Lane Health Centre, Sutton, London, UK; 2 Imperial Clinical Analytics Research & Evaluation (iCARE) Secure Data Environment, NIHR Imperial Biomedical Research Centre, Imperial College Healthcare NHS Trust, London, UK; 3 Faculty of Medicine, Department of Surgery and Cancer, Imperial College London, London, UK; 4 NIHR North West London Patient Safety Research Collaboration, Imperial College London, London, UK; 5 Imperial College Healthcare NHS Trust, London, UK

**Keywords:** Electronic Health Records, Safety, Information Storage and Retrieval, Hospitals, Quality in health care, Health & safety

## Abstract

**Abstract:**

**Introduction:**

Manual investigation of falls incidents for quality improvement is time-consuming for clinical staff. Routine care delivery generates a large volume of relevant data in disparate systems, yet these data are seldom integrated and transformed into real-time, actionable insights for frontline staff. This protocol describes the co-design and testing of a safe mobility and falls informatics platform for automated, real-time insights to support the learning response to inpatient falls.

**Methods:**

Underpinned by the learning health system model and human-centred design principles, this mixed-methods study will involve (1) collaboration between healthcare professionals, patients, data scientists and researchers to co-design a safe mobility and falls informatics platform; (2) co-production of natural language processing pipelines and integration with a user interface for automated, near-real-time insights and (3) platform usability testing. Platform features (data taxonomy and insights display) will be co-designed during workshops with lay partners and clinical staff. The data to be included in the informatics platform will be curated from electronic health records and incident reports within an existing secure data environment, with appropriate data access approvals and controls. Exploratory analysis of a preliminary static dataset will examine the variety (structured/unstructured), veracity (accuracy/completeness) and value (clinical utility) of the data. Based on these initial insights and further consultation with lay partners and clinical staff, a final data extraction template will be agreed. Natural language processing pipelines will be co-produced, clinically validated and integrated with QlikView. Prototype testing will be underpinned by the Technology Acceptance Model, comprising a validated survey and think-aloud interviews to inform platform optimisation.

**Ethics and dissemination:**

This study protocol was approved by the National Institute for Health Research Imperial Biomedical Research Centre Data Access and Prioritisation Committee (Database: iCARE—Research Data Environment; REC reference: 21/SW/0120). Our dissemination plan includes presenting our findings to the National Falls Prevention Coordination Group, publication in peer-reviewed journals, conference presentations and sharing findings with patient groups most affected by falls in hospital.

**STRENGTHS AND LIMITATIONS OF THIS STUDY**

- This multicentre study brings together a learning community of clinical and non-clinical staff from diverse specialities and professional groups to collaborate with patients, data scientists and researchers to co-design a safe mobility and falls informatics platform—the *insightFall* platform.
- Our co-production approach is underpinned by human-centred design principles to understand and address end-user needs and to ensure clinical utility of the informatics platform at individual user and organisational levels.
- The platform will use natural language processing to harness routinely collected data from electronic health records and incident reports and will display data-driven insights in an accessible way for clinical staff and safety improvement teams.
- The accuracy and completeness of routine falls documentation are likely to limit the quality of data-driven insights that can be delivered.

## Introduction

### Overview

 Among the most frequently reported patient safety incidents, hospital falls remain a significant challenge for healthcare services, specifically causing physical and psychological harm to patients and negatively affecting their health, independence and quality of life.^1^,^4^ Monitoring and investigating why falls occur is essential for quality improvement, but the current manual process of extracting insight from clinical documentation is time-consuming, with learning often taking place in siloes.^5^ High quality (timely, complete, accurate) data are enablers of patient safety but healthcare services continue to be underserved by effective information systems that provide clinical teams with meaningful and actionable insight.^6^,^10^

### Safety incident data

The core data of patient safety incident reporting systems are the reports submitted by clinical staff; deeper insight into contributing factors can be obtained through additional information gathering—from patient records and other hospital systems, and from patients, families and healthcare professionals. These insights should prompt improvement activity such as the redesign of local policy or changes to care processes towards reducing risk for future patients, but the ability to affect positive change requires social capital (learning communities, improvement capability, culture) and technical infrastructure (data, tools, systems, analytic capability).^11^ Effective learning systems are therefore challenging to operationalise, and few health systems have achieved this cycle of data-driven improvement.^10 11^ The reasons for this are often logistical: documentation is held in disparate systems, data extraction and interpretation are still manual, ward-level processes, and investigators are usually clinical leads with multiple competing commitments.^10 12 13^ Accordingly, the recent WHO report indicates that many incidents remain unaddressed, and safety incident data are frequently wasted.^10^

### Learning from patient safety events

In the context of the English National Health Service (NHS), NHS England’s new Patient Safety Incident Response Framework (PSIRF) intends to shift the balance from the collation of exhaustive investigation reports that seldom lead to meaningful change, to embedding the patient safety incident response within a culture of improvement.^14^ NHS organisations will be expected to adopt a coordinated and data-driven approach to understanding their patient safety incident profiles; however, optimal strategies for achieving this are yet to be realised.^14^ While the last two decades have seen widespread uptake of electronic records, the potential of these systems to enhance patient safety through the use of routinely collected data has not been fully exploited.^13 15 16^ In recent years, the volume of clinical information accumulating in electronic systems has increased exponentially, offering a unique ‘digital information fingerprint’ of the complex healthcare system in which our patients are cared for.^13 16 17^ Clinical documentation contains rich information about patient symptoms, diagnoses, comorbidities, medications, investigations, results and treatment; operational metrics comprise staffing, bed occupancy, waiting times, length of stay and patient experience—and these lists are certainly not exhaustive. National clinical audits represent large-scale data resources accessible to clinical teams, managers, policy makers, patients and researchers for the purpose of driving improvement in care quality.^18^ The National Audit of Inpatient Falls (NAIF) aims at improving falls risk reduction practice by collating data on the delivery and quality of care for inpatients over 65 who fall and sustain a hip or femoral fracture in England and Wales, and by communicating findings through the annual NAIF report.^19^ Although senior healthcare professionals consider NAIF insights and recommendations to be useful, they have also emphasised the value of continuous monitoring and feedback on falls, with better engagement with frontline staff who are best-placed to deliver improvement.^18^ Foy and colleagues argue that the impact of audit could be maximised through monitoring systems designed to reduce administrative workload burden of collating data.^13^ Without effective information systems, healthcare organisations and clinical teams are limited in their ability to identify important trends, direct improvement activity or systematically and iteratively evaluate the impact of improvement initiatives.^9^

### Implementing learning health systems

**Figure 1:**
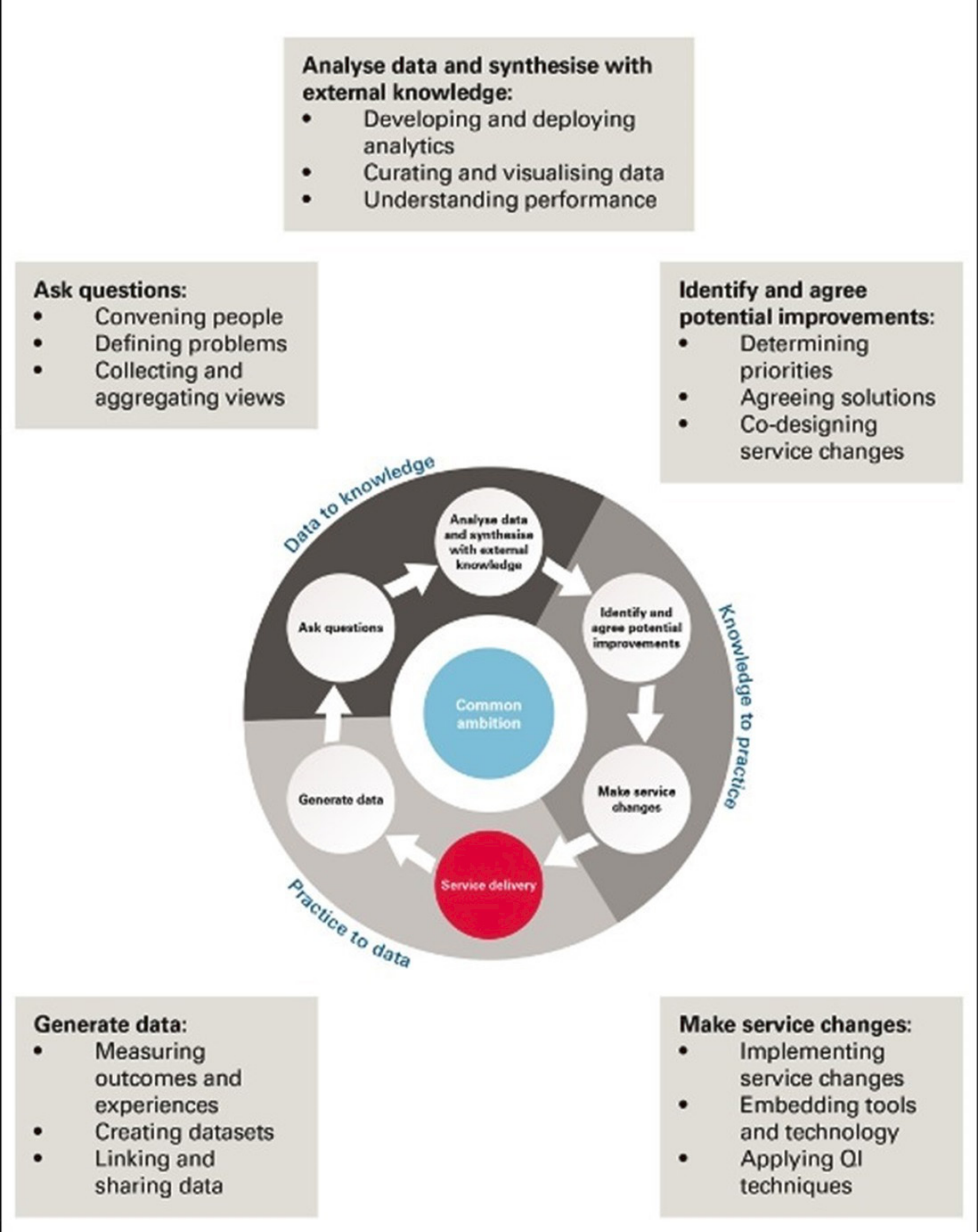
Learning health systems: stages and activities of the learning and improvement cycle. Source: The Health Foundation.^11^ QI, Quality Improvement.

Learning health systems are able to learn from the routine care they deliver as part of business as usual, using a systematic approach to improving quality and safety through continuous cycles of information, actions and improvement (figure 1).^11^ The formation of a learning community of people with different roles and expertise (healthcare professionals, patients, carers, data analysts, researchers), with responsibility for identifying problems and implementing data-driven improvement, is pivotal to the success of the learning health system approach.^11 12 19 20^

### Practice-to-data

The learning cycle begins with data generated during routine service delivery. Patient data must be collected, stored and used for patient and health service benefit in a way that earns public trust through security and transparency; therefore, secure data environments (SDEs) are becoming the default way to access health and social care data for research and innovation in the NHS.^621^,^23^ SDEs are data storage and access platforms, which maintain the highest standards of privacy and security by allowing approved users to access and analyse data without the data leaving the secure environment.^23^ Learning communities must clearly define the data relevant to the problem of interest and may work with data curation experts to extract, clean and transform raw data into a format that can be used for evidence generation.

### Data-to-knowledge

Large volumes of data are collected during routine care delivery, but data are rarely available to clinical teams in a format timescale that allows it to be useful for improvement; data science offers the realistic potential of addressing this issue.^24^,^26^

#### Natural language processing

Natural language processing (NLP) is a branch of artificial intelligence (AI) that seeks to process and interpret human language using computer-based algorithms to capture meaning and identify relationships.^24^ Clinical records and incident reports contain both structured and unstructured data: structured information fields include discrete data such as age, sex, ward location, vital signs and level of harm; unstructured data are any free-text narrative documentation with the electronic record that can describe a patient’s condition, the events leading to an incident and contributory factors (such as communication failures or medication side-effects). While reporting systems do incorporate structured data fields that permit incident classification, reporters rarely use checkbox options to indicate contributory factors because the taxonomies seldom reflect the complex interplay of factors leading to an incident.^27^ Thus, a substantial portion of reported information exists in free-text format and due to the time-consuming nature of triangulating and interpreting free-text information, these free-text data remain largely under-used for incidents not causing harm. NLP can address these challenges by enhancing understanding of the multifactorial causes of safety incidents in an automated way. Studies have explored the application of NLP for adverse events analysis,^1724 27^,^29^ including incident classification (type and severity) and extraction of contributory factor information,^24 27^ and to examine how patients, nurses and the environment interact to cause falls.^29^

#### Delivering data-driven insight to clinical teams

Methods to visualise patient safety data can support clinical teams to better understand their safety problems.^9^ A small number of patient safety dashboards have integrated NLP models but while evidence has demonstrated the potential of NLP to support safety improvement, most have focused on model performance rather than evaluating impact in routine service delivery.^30^,^32^ A key criticism of existing safety dashboards is the failure to incorporate human factors principles into their design and evaluation.^8^ Human-centred design focuses on developing an understanding of people and their needs, involves stakeholders early and throughout the design process and considers how an intervention will be successful at both the individual user level and at the level of the organisation.^33^ The utility of clinical analytics can be optimised through collaborative approaches involving patients, healthcare professionals and data scientists to co-design analytic pipelines.^34^

### Knowledge-to-practice

Discussions of learning health systems sometimes infer a somewhat automated sequence of practice-to-data, data-to-knowledge and knowledge-to-practice, but central to effective learning cycles are the social processes of defining problems, setting priorities, and co-designing and implementing service changes.^11^ Effective collaboration between members of the learning community to interpret data-driven insight, gradual refinement of an intervention based on repeated tests of change and skills in managing complexity and leading change are essential for successful and sustainable quality improvement in routine practice.^35^ Bringing together a learning community with a focus on integrating local data insights with robust evidence could optimise workforce planning and processes towards safe mobility. Research demonstrates that patient and staff education and multifactorial interventions can reduce hospital falls^2 36 37^; easily accessible local falls data could help to ensure that education programmes and falls prevention strategies are tailored to mitigate prevalent falls risks in different inpatient settings.

### Rationale

Most healthcare systems are characterised by the concept of ‘data rich, information poor’, with data held in silos, rarely integrated to produce meaningful and actionable insights for clinical staff.^9^ Manual data collection can be burdensome for frontline clinical professionals, taking them away from the critical task of delivering healthcare.^6 22^ Inpatient falls occur frequently and require a learning response by healthcare professionals. By moving away from manual data collection towards automated approaches, clinical teams can spend more time identifying solutions and delivering improvement.

## Study aim

This study aims to co-produce and formatively evaluate a safe mobility and falls informatics platform in the acute hospital setting to provide automated, real-time insight into inpatient falls. We hypothesise that readily available data on the context, circumstances, mechanisms and outcomes of falls will enable meaningful quality improvement activity to reduce injurious falls in the inpatient setting.

### The *insightFall* platform

The informatics platform is intended to (1) provide data-driven insight to support the initial learning response when a fall occurs, (2) guide meaningful, coordinated quality improvement activity based on trends in aggregated data and (3) provide a mechanism for near-real-time monitoring of inpatient falls at Trust-level (assurance). Intended users of the *insightFall* platform are ward managers, matrons and the Safe Mobility and Falls Prevention specialist clinical team, and Safe Mobility and Falls Prevention Steering Committee. This work is an important first step towards operationalising a learning health system for safe mobility and falls prevention in secondary care.

## Methods and analysis

### Study design

**Figure 2:**
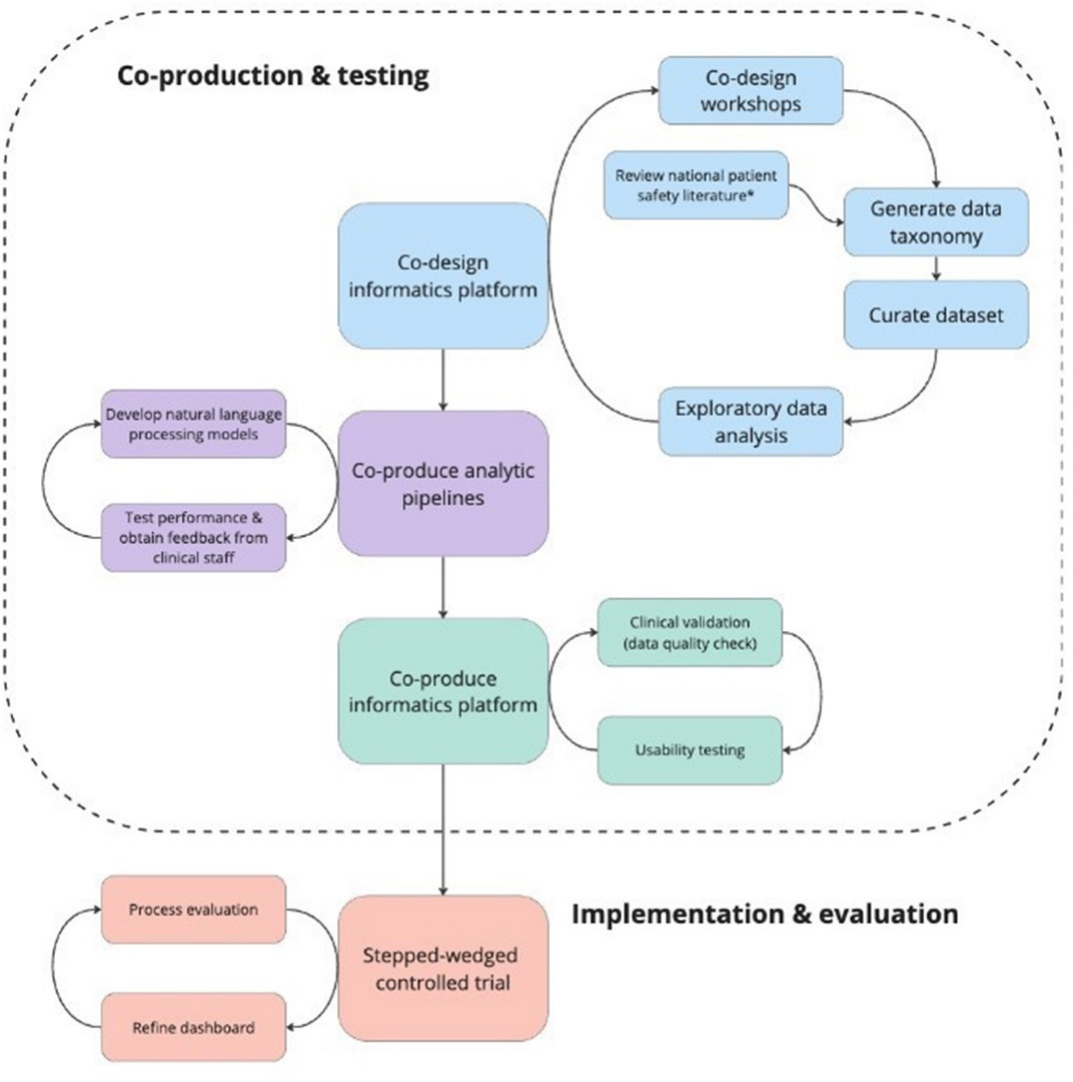
Study overview. Scope of current study proposal denoted by the dashed line. *National Adult of Inpatient Falls (NAIF) & the Patient Safety Incident Response Framework (PSIRF).

The current study comprises three overall components, underpinned by human-centred design principles and co-production: (1) co-design of the *insightFall* platform with patients and clinical staff, (2) integration of data sources and development of NLP algorithms and pipelines and (3) prototype usability testing with end-users (figure 2).

### Study population and setting

Imperial College Healthcare NHS Trust, a large acute care provider in North-west London, is using routine data and advanced clinical analytics to support improvements in patient safety and care quality. De-identified data from electronic patient records and the incident reporting system are held within the Trust’s SDE ‘Imperial Clinical Analytics, Research and Evaluation’ (iCARE) providing the secure data infrastructure and specialist analytical and data science capabilities for the implementation of learning health systems. A learning community of approximately 30 clinical and non-clinical staff from diverse specialities and professional groups (nursing staff, therapists, doctors, ward managers, matrons, the safe mobility and falls prevention steering committee and specialist clinical team, and the patient safety team) will work with data scientists and researchers to co-design the *insightFall* platform. Opportunities to contribute to the co-design of the platform will be circulated via Trust management meetings and mailing lists, with managers being asked to cascade the opportunities to staff in their areas (snowball sampling). Staff interested in being involved will be invited to contact the project team by email. We will monitor the number and professional background (role, clinical specialty) of Trust staff contributing to the co-design.

### Patient and public involvement, engagement and participation (PPIEP)

Previous PPIEP to inform the development of research themes within the National Institute for Health Research Imperial Biomedical Research Centre demonstrated strong public support for using AI to maximise the learning from patient data.^38^ This project’s co-production approach embeds PPIEP at all stages and a diverse panel of lay partners will be involved throughout the project life cycle. Two lay partners (patient representatives) will be appointed to the project steering group to provide oversight for the acceptable use of de-identified patient data towards a learning health system for safe mobility and falls prevention. Three additional lay partners will contribute and participate alongside healthcare professionals in the co-design sessions to ensure that data and insights that matter to patients are incorporated into the design of *insightFall* platform.

### Co-design of the *insightFall* platform

#### Overview

Design of the safe mobility and falls informatics platform will draw on evidence of the features of effective patient safety dashboards, clinical informatics and human-centred design principles.^8 33^ Data to be displayed in the platform will be informed by NHS England’s new PSIRF,^14^ the National Audit of Inpatient Falls,^39^ national and international guidance on assessing falls risk and prevention,^3 40^ and co-design workshops with patients and end-users of the dashboard.

#### Co-design

Co-design ensures that contextual factors are taken into account, facilitates the uptake of the intervention and user satisfaction.^33 41^ The first round of co-design workshops will explore the needs and preferences of patients and end-users of the informatics platform. There will be one workshop at each of the three main hospital sites; each workshop will involve up to 15 participants, including clinical staff, patient safety team members and patient representatives. Workshop activities will include small group discussions to explore: (1) current processes of collecting and using falls data (what works and what are the challenges), (2) perspectives on the data that should be displayed in a safe mobility and falls informatics platform and (3) how the data should be visualised. The co-design process will be underpinned by the Double Diamond framework—a process of exploring problems and solutions widely (divergent thinking) followed by considering the issues in a more focused way (convergent thinking).^42^ For example, end-users’ data requirements will be determined through a process of ‘blue-sky thinking’ to consider all possible data items, followed by a prioritisation activity to categorise data items into ‘essential data’ (must be included), ‘desirable data’ (would be good to include) and ‘aspirational data’ (data considered to be important that is not currently collected or documented electronically). Participants will use sticky notes and chart paper to document individual perspectives and group discussion points. Additionally, a patient safety researcher with qualitative and ethnographic expertise (PA) will take detailed notes to capture group discussions and verbatim quotes from participants. Content analysis will be used to derive descriptive categories reflecting current processes for collecting and using falls data and perspectives on the data elements for the new falls informatics platform. A summary report will be written and circulated to participants for comment.

#### Data elements

The data requirements for the *insightFall* platform will be specified by clinical staff during the co-design workshops. Data elements will likely include:

- Falls rates (of note, since raw numbers of falls can be misleading due to variation in bed occupancy and length of stay, rates can be expressed as falls per 1000 occupied bed days).^43^
- Data from multifactorial risk assessments and care plans (including the risk factors identified, the interventions put in place to mitigate falls risk, and how frequently assessments and care plans were updated by clinical staff).^3 40^
- Contextual information (eg, time and location of falls).
- Post-falls management (eg, medical assessment within 30 min).^39^
- Outcomes (eg, rate of femoral fractures).^39^

The platform will not display metrics on risk categories; Falls Risk Assessment Tools that assign risk scores and place patients in risk categories (eg, ‘no risk, ‘low risk’, etc) or are not used in our Trust since robust trials and national guidelines do not support their use.^40 44^

#### Exploratory data analysis

Based on the co-designed data taxonomies, a historical static dataset (1 November 2022 to 30 April 2023) will be curated comprising de-identified, linked, routine data from electronic patient records (Cerner) and incident reports (Datix) (approximately 750 falls). Since these data are collected as part of routine care delivery and not for the specific purposes of this project, the properties of the data will be explored to determine how they can be transformed into actionable insight.^45^ The structure of the data (variety) will be examined to determine whether summary statistics or free-text analysis will be needed. The quality (veracity) of the data will be assessed to determine the proportion of missing, noisy or inconsistent data for each variable.^46^ This stage of data pre-processing is important since real-world electronic health record data are frequently ‘messy’ due to erroneous or incomplete clinical documentation.^46^ Preliminary insights related to the context, circumstances, mechanisms and outcomes of falls will be extracted and presented alongside the percentage of missing or erroneous values per variable (values outside the expected parameters—eg, Glasgow Coma Scale of 19). These data will be reviewed by and discussed with the Trust’s falls prevention team to understand their potential value for measuring and monitoring the problem of inpatient falls and for informing quality improvement activity.

#### Prototype development

The platform prototype will be developed by incorporating the goals and data requirements specified by clinical staff as the end-users and an understanding of the availability and quality of the data. The initial wireframes (high-level platform components) will be presented during the Trust’s annual Falls Summit, and feedback will be gathered from around 50 clinical staff (using Mentimeter) to reach consensus on the data elements to be displayed in the *insightFall* platform. A second round of co-design workshops with end-users will focus on how these data should be displayed. The data will be presented in a variety of ways (tables, visualisations), and end-users will rate the insights based on how easy they are to understand. In the final stage of development, a data developer will create the initial prototype for user testing in Qlik Sense.

### Co-production of analytic pipelines

NLP pipelines will be developed to provide automated, near-real-time insights into the causes, circumstances and mechanisms of patient falls. The analytic pipelines will first clean and then analyse free-text data, and the outputs will be displayed in QlikView (the user interface) (table 1). Domain knowledge (clinical expertise) will be incorporated at various stages of algorithm development through an iterative process of developing, clinically verifying and refining the models. First, a core advisory group of clinical staff will define the initial lexicon of terms (vocabulary) that represent key concepts related to falls (eg, ‘zimmer frame’, ‘walking stick’, ‘gutter frame’ are terms related to the concept ‘mobility aids’). These vocabularies will be used to build data extraction rules for the NLP models. The rules will be applied to free-text narratives in our static dataset to generate a corpus of annotated incident reports and falls documentation, with the concept classes ‘tagged’ by the NLP model. A second stage of clinical input will task the clinical advisory group with reviewing the annotated documents to assess the extent to which the NLP models are generating clinically meaningful outputs. Where tagging is erroneous or inconsistent, the informatics team will adapt and refine the model to improve its performance and clinical relevance. The process of clinical verification and model refinement will continue until the advisory group agrees that model outputs provide clinically useful insight. As a final step, the NLP models will be applied to the most recent 1 year of unseen falls documentation. The advisory group will manually tag the concept classes without viewing the predicted classes to avoid bias, and inter-rater agreement will be calculated at the note level to appraise the level of agreement. To evaluate the overall performance of the NLP models, the F1 score will be calculated for each label. Previous work has shown that rule-based models applied to falls documentation can achieve an F1 score of at least 70%.^47 48^ Given the specific requirements of the clinical application, this will be set as the target before the model is deployed for real-time use. Once the NLP models have been validated, their outputs will be integrated with QlikView and displayed in accordance with the preferences of clinical staff.

**Table 1:** Developing a natural language processing model

*Definition*	
Natural language processing (NLP)	Computer-based algorithms (instructions) that enable insights to be derived from unstructured clinical text by extracting, codifying and storing it in a structured format for downstream analysis.
*Steps involved in developing an NLP model*	
Understanding and building a corpus of words	It is important to first understand the vocabulary that the unstructured clinical notes may contain, and which words can be used to extract insights. These taxonomies are derived from clinically validated sets of terms through consultation with clinical staff and review of relevant documentation.
Cleaning the text	To ensure the quality, reliability and effectiveness of the subsequent analysis, it is important to standardise the text reducing variations in the structure. This involves techniques like expanding clinical abbreviations, correcting spelling mistakes, stemming or lemmatising (reducing similar words to a common word) and removing stop words (commonly used words such as ‘and’, ‘the’, ‘is’).
Information extraction	To gain insights from text, various techniques can be used^54^ Named entity recognition: identifies and classifies words in a text (named entities) into categories (eg, person, location). Relation extraction: extracts associations between the meaning of words connecting two specified named entities in a sentence.

### Prototype usability testing

Clinical staff will be invited to participate in usability testing, comprising of audio-recorded think-aloud interviews and completion of the System Usability Questionnaire.^49 50^ An email inviting staff to participate in usability testing will be sent to previous workshop participants, with further recruitment via Trust management meetings and mailing lists. Previous co-design workshop participants and clinical managers will be asked to cascade the interview opportunity to their colleagues (snowball sampling). Staff willing to be interviewed will be asked to contact the researcher by email; written informed consent will be sought for participation. A sample of 20 staff members with diverse professional characteristics (professional role, clinical specialty or directorate, work location) will be recruited. Staff will have the opportunity to openly interact with the dashboard and will receive verbal prompts from the interviewer, for example, ‘What are your first impressions about the platform? What are you thinking now? What makes you think that?’ The prompts will become more task focused, for example, ‘How might you use the platform to support you with your incident response documentation after a patient has fallen? Talk me through it. How might you use the dashboard to support a quality improvement project around falls in your ward? Show me which data you would look at’. After the think-aloud interview, participants will complete the System Usability Scale questionnaire.^49^ The System Usability Scale is a validated, 10-item questionnaire with Likert-type responses that gives a global view of end-users’ subjective assessments of usability (eg, ‘I thought the system was easy to use’; ‘I think I would need the support of a technical person to use this system’).^49^

Qualitative data from the audio-recorded think-aloud interviews will be transcribed verbatim and analysed within NVivo software using Framework Analysis,^51^ drawing on the Technology Acceptance Model as a preliminary framework.^52^ Two researchers (RLe, PA) will carry out the Framework Analysis according to the five-step analytical approach described by Ritchie and Spencer,^51^ which permits systematic and comprehensive analysis of qualitative data generated within applied research. The researchers will begin with a process of familiarising themselves with transcribed data, making notes as to recurrent ideas and concepts to shape the identification of an initial thematic framework. Addressing our aim to understand the usability of the informatics platform, the initial thematic framework will be informed by concepts derived a priori from the Technology Acceptance Model,^52^ including ‘perceived usefulness’, ‘perceived ease of use’ and ‘behavioural intention to use’, together with additional themes arising from study data. The thematic framework will be systematically applied to all transcripts, with data abstracted, summarised and synthesised into a framework matrix in a process termed ‘charting’. A final interpretation stage will encompass characterisation of the range and nature of participant perspectives on system usability, with identification of ways in which prototype usability could be optimised to enhance user experience and intervention uptake. Questionnaire responses will be summarised using descriptive statistics. The prototype usability testing will inform refinements to the platform before implementation and summative evaluation.

### Evaluating the quality of the coproduction approach

As recommended by Nordin and colleagues,^53^ our co-production approach will be evaluated from three perspectives: (1) the outputs of co-production processes, (2) participants’ experiences of participating in co-production and the (3) outcomes of co-production processes. The outputs of the co-design workshops and usability testing will consist of three insights reports: the first report will outline the data elements that clinical staff would like to see displayed in the *insightFall* platform; the second report will outline how clinical staff would like to see data-driven insights displayed (including their preferences for different types of visualisations); the final report will bring together System Usability Scale results with the think-aloud findings aligned with the Technology Acceptance Model (perceived usefulness, perceived ease-of-use and intentions to use the platform).^49 52^ Participants’ experiences of co-production will be evaluated using a two-item web-based questionnaire administered using Microsoft Forms at the end of each co-design workshop; item 1 is an overall workshop rating (1–5 stars) and item 2 asks participants to justify the rating they gave. The questionnaire was designed to be short and simple based on feedback that completion of the questionnaire should not be burdensome for busy clinical staff who have already given up time to participate in the co-design session. Co-production outcomes will be evaluated using the following metrics: (1) the proportion of end-users with Systems Usability Scores >68 in usability testing and (2) the proportion of wards and directorates with sustained adoption at the end of the implementation and evaluation phase.

### Implementation and evaluation

The co-produced *insightFall* platform will be incorporated into Imperial College Healthcare NHS Trust’s patient safety incident response plan and policy, in line with NHS England’s PSIRF.^14^ Reviewing data-driven insights on the informatics platform will be built into the incident response workflow for falls, enabling ward managers and matrons to incorporate data on the context, circumstances, mechanisms and outcomes of falls into ward-level safety improvement activity without the burden of manually collating data from clinical records. At Trust-level, learning from the platform will be built into the cycle of business and work of the Safe Mobility and Falls Prevention Steering Committee and specialist clinical team. Trust, Divisional and Directorate-level data insights will used for quality assurance, and to direct resource towards improvement efforts to priority clinical areas. Reporting a detailed evaluation plan is outside the scope of the current protocol, however, future plans include testing the feasibility of the data-driven quality improvement approach using a mixed-methods stepped-wedged design with rapid-cycle process evaluation to create a regular feedback loop between end-users and the informatics team to enable the platform to be refined during implementation. Trial endpoints will include the uptake and use of the *insightFall* platform (Qlik Sense audit metrics), quality improvement activity related to safe mobility and falls (Life QI metrics) and rates of injurious falls.

## Ethics and dissemination

This study uses de-identified patient data within the Imperial Clinical Analytics Research and Evaluation (iCARE) SDE; iCARE is an NHS Research Ethics Committee approved database that is hosted entirely within Imperial College Healthcare NHS infrastructure (REC reference: 21/SW/0120). The system pulls data from clinical systems used in care within the hospital, processes, and curates these into data that can be used in research, evaluation, audit and clinical analytics. Patient consent is not sought since the data in the iCARE environment are collected as part of routine care delivery; no patient data will be collected specifically for this study, and no patient identifiable data will be seen by researchers. Imperial College Healthcare NHS Trust’s General Data Protection Regulation (GDPR) compliant Privacy Policy (made available to all patients via posters and the internet) explains that data collected during care will be de-identified within the NHS and will be used for care improvement, audit, evaluation and research. The iCARE system operates a user to data model (with researchers accessing data on NHS Trust Infrastructure). All data access requests are reviewed by the Imperial Data Prioritisation, Access and Ethics Committee, which provisions ethical access to the de-identified research database based on the overarching NHS Research Ethics Committee database approval. The Access Group, which includes senior clinicians and researchers, members of the Trust’s Data Protection Office, the Caldicott Guardian and lay partners, reviews and recommends data access for projects based on clinical needs and patient and public interest.

Our dissemination plan includes presenting our findings to the National Falls Prevention Coordination Group, publication in peer-reviewed journals, conference presentations, and sharing findings with patient groups most affected by falls in hospital.
