# Supplementary material for: Co-producing a safe mobility and falls informatics platform to drive meaningful quality improvement in the hospital setting: a mixed-methods protocol for the insightFall study
Source: BMJ Open. 2025 Feb 3;15(2):e082053. doi: 10.1136/bmjopen-2023-082053 (PMC11795406; doi:10.1136/bmjopen-2023-082053)
Supplement: Supplementary file 1 [file bmjopen-2023-082053.reviewer_comments.pdf]

PEER REVIEW HISTORY

BMJ Open publishes all reviews undertaken for accepted manuscripts. Reviewers are asked to complete a checklist review form and are provided with free text boxes to elaborate on their assessment. These free text comments are reproduced below.

ARTICLE DETAILS

Title (Provisional)

Co-producing a safe mobility and falls informatics platform to drive meaningful quality improvement in the hospital setting: a mixed-methods protocol for the insightFall study

Authors

Lear, Rachael; Averill, Phoebe; Carenzo, Catalina; Tao, Rachel; Glampson, Ben; Leon-Villapalos, Clare; Latchford, Robert; Mayer, Erik

---

VERSION 1 - REVIEW

**Reviewer**	**1**
**Name**	**Morris, Meg**
**Affiliation**	**La Trobe Univ, Room 423 HS3**
**Date**	**16-Feb-2024**
**COI**	**Nil**

This novel, engaging and well written manuscript addresses the under-researched topic of how best to document and report hospital falls.

1. More specific details are needed on the type of data that needs reporting, especially falls rates per 1000 bed days, injury rates per 1000 bed days, falls risk (various ways of measuring), and sentinel events pertaining to falls. Sometimes staff simply document the number of falls which does not take into account length of stay or bed occupancy. Please see (and cite) the world falls guidelines for examples:

Montero-Odasso, M., (2022). World guidelines for falls prevention and management for older adults: a global initiative. Age and Ageing, 51(9).

2. The manuscript could give more details to capture clinical data collection stages and accompanying data needed: (screening for falls on day 1, comprehensive falls assessment for older patients and those with multi-morbidity, 3 interventions to prevent and mitigate falls) and related issues re these stages , such as whether or not to collect FRAT data using falls risk assessment tools - for example see Morris, M. E., (2021). Divesting from a Scored

Hospital Fall Risk Assessment Tool (FRAT): A Cluster Randomized Non-Inferiority Trial. Journal of the American Geriatrics Society, 69(9), 2598-2604.

3. It seems that a waiver of consent will be requested - there needs to be a clear ethics statement about why a waiver is needed so confidential de-identified patient data can be gathered from the system without the knowledge of patients.

4. In the background and discussion the commentary could refer more to articles on hospital falls prevention, especially referring to patient and staff involvement in providing data and using data for evidence based practice and workforce redesign & processes. Some examples could include:

Heng, H., (2022). Healthcare professional perspectives on barriers and enablers to falls prevention education: A qualitative study. PLoS ONE, 17(4 April).

Morris, M. E., T (2024). Preventing hospital falls: feasibility of care workforce redesign to optimise patient falls education. Age and Ageing, 53(1), 9 pages.

Shaw, L. K. (2021). Improving hospital falls screening and mitigation using a health professional education framework. Nurse Education Today, 98.

**Reviewer**	**2**
**Name**	**Choi, Yoona**
**Affiliation**	**Seoul National University**
**Date**	**28-Feb-2024**
**COI**	**No competing interests.**

Review Checklist Q1, 3, 4.

- The project outline is commendable and demonstrates a clear ambition to contribute meaningfully to the field. However, it could benefit from a more explicit articulation of its specific aims and hypotheses.

- The protocol appears overly descriptive in parts, without adequately specifying what will be investigated. Clarifying the research questions and the variables or outcomes of interest would greatly enhance the study's focus and impact.

- There is a need for more detailed descriptions of the data elements that will be used to investigate these variables or outcomes. For instance, when discussing information extraction, it would be helpful to specify the types of information being sought (e.g., number of falls, timing of falls, events occurring before or after falls).

- It would be beneficial to outline the expected outcomes more clearly. What insights does the study aim to provide? If the goal is to identify patterns in patient characteristics or situations leading to falls, how will this information be used?

- If the study intends to develop a platform based on its findings, how will this platform be utilized? For example, is there an aim to alert medical staff to potential fall risks preemptively? Or just to gather related insights? Clarifying the intended applications of the research findings could significantly enhance the protocol's relevance and applicability.

Review Checklist Q 6, 7.

- The abstract mentions exploratory analysis of variety, veracity (accuracy/completeness), and value, yet lacks detailed descriptions of how these metrics are defined and evaluated. Clarification on the methodologies for measuring and analyzing each would provide greater insight into the study's objectives and potential impacts.

- Regarding the study population and setting, it's mentioned that de-identified data from electronic patient records will be used. However, specifics on the time frame for data collection, the patient population from which the data will be extracted, etc. are missing. Additionally, an approximate sample size for both patients and participating medical staff should be stated to give a sense of scale and scope.

- In the section on prototype usability testing, criteria for what constitutes "usability" are not defined. It would be beneficial to specify what conditions or benchmarks will be used to evaluate usability. Furthermore, recommendations on the appropriate number of staff members to be involved in usability testing would help in understanding the expected reliability and applicability of the testing outcomes.

Reviewer **3**

Name **Ludlow, Kristiana**

Affiliation **Macquarie University, Australian Institute of Health Innovation**

Date **01-Mar-2024**

COI **I have no competing interests.**

This is a well-written manuscript that was clear and engaging. A good overview of previous literature and background context was provided, as well as a strong study rationale. The methods were clearly described; however some details were missing. My feedback to further strengthen the manuscript is below.

1. Can you please explain who “lay partners” might be, e.g., patients, caregivers, members of the public?

2. What activities will the co-design workshops involve? For example, you say “the written content of co-design activities”, but it’s not clear what activities will be conducted beyond verbal discussions.

3. Please provide a detailed account of the qualitative data analysis, e.g., analysis steps, who will be involved, software etc.

4. “Content analysis will be used to derive descriptive categories and themes”. Content analysis identifies categories, whereas thematic analysis identifies themes. Will you be using both?

5. How will you move from co-design to usability testing? It seems a step around prototype development is missing.

6. How many participants will be involved in the usability testing and co-design workshops?

7. Will participants be openly interacting with the dashboard during usability testing or assigned tasks?

8. Will participants be given the opportunity to engage in think-aloud before being interviewed? The manuscript currently reads as an interview during a task, rather than think-aloud.

9. An evaluation of the co-design approach will be conducted through web-based questionnaires. What are these questionnaires and how will data be analysed?

10. Could you please provide more information about: “Secondary analysis of data collected as part of the trust’s Ward Accreditation Programme and trust-wide administration of the research impact capture tool will also contribute towards the evaluation”.

11. Do you have plans to implement and evaluation the platform? While this might be outside the scope of the co-design study, adding some brief information about future plans would be beneficial.

12. Please provide a reference(s) for the sentence starting with: “These insights should prompt improvement activity such as the redesign …”

13. Please provide a reference for the sentence: “Methods to visualise patient safety data can support clinical teams to better understand their safety problems.”

VERSION 1 - AUTHOR RESPONSE

Reviewer: 1

Prof. Meg Morris, La Trobe Univ

Comments to the Author:

This novel, engaging and well written manuscript addresses the under-researched topic of how best to document and report hospital falls.

RESPONSE: Many thanks for this positive feedback and for your valuable suggestions to improve the manuscript, which we have now addressed as detailed below.

1. More specific details are needed on the type of data that needs reporting, especially falls rates per 1000 bed days, injury rates per 1000 bed days, falls risk (various ways of measuring), and sentinel events pertaining to falls. Sometimes staff simply document the number of falls which does not take into account length of stay or bed occupancy. Please see (and cite) the world falls guidelines for examples:

Montero-Odasso, M., (2022). World guidelines for falls prevention and management for older adults: a global initiative. Age and Ageing, 51(9).

2. The manuscript could give more details to capture clinical data collection stages and accompanying data needed: (screening for falls on day 1, comprehensive falls assessment for older patients and those with multi-morbidity, 3 interventions to prevent and mitigate falls) and related issues re these stages , such as whether or not to collect FRAT data using falls risk assessment tools - for example see Morris, M. E., (2021). Divesting from a Scored Hospital Fall Risk Assessment Tool (FRAT): A Cluster Randomized Non-Inferiority Trial. Journal of the American Geriatrics Society, 69(9), 2598-2604.

RESPONSE: Thank you for suggesting that we include further details on the type of data to be reported. In response to your feedback we have added the following paragraph under the sub-heading ‘Data elements’; we have referenced the two citations you suggested as well as our UK national falls clinical and reporting guidelines:

Data elements

The data requirements for the insightFall platform be specified by clinical staff during the co-design workshops. Data elements will likely include:

• falls rates (Of note, since raw numbers of falls can be misleading due to variation in bed occupancy and length of stay, rates can be expressed as falls per 1000 occupied bed days [43])

• data from multifactorial risk assessments and care plans (including the risk factors identified, the interventions put in place to mitigate falls risk, and how frequently assessments and care plans were updated by clinical staff [3,40])

• contextual information (e.g. time and location of falls)

• post-falls management (e.g. medical assessment within 30 minutes [39])

• outcomes (e.g. rate of femoral fractures [39]).

The platform will not display metrics on risk categories; Falls Risk Assessment Tools that assign risk scores and place patients in risk categories (e.g. 'no risk, 'low risk', etc.) or are not used in our Trust since robust trials and national guidelines do not support their use [40,44].

3. It seems that a waiver of consent will be requested - there needs to be a clear ethics statement about why a waiver is needed so confidential de-identified patient data can be gathered from the system without the knowledge of patients.

RESPONSE: We have expanded the section on 'ethics and dissemination' to provide details about why consent was not sought for use of patient data:

This study uses de-identified patient data within the Imperial Clinical Analytics Research and Evaluation (iCARE) secure data environment; iCARE is an NHS Research Ethics Committee approved database that is hosted entirely within Imperial College Healthcare NHS infrstructure (REC reference: 21/SW/0120). The system pulls data from clinical systems used in care within the hospital, processes, and curates these into data that can be used in research, evaluation, audit and clinical analytics. Patient consent is not sought since the data in the iCARE environment are collected as part of routine care delivery; no patient data will be collected specifically for this study and no patient identifiable data will be seen by researchers. Imperial College Healthcare NHS trust's GDPR compliant Privacy Policy (made available to all patients via posters and the internet) explains that data collected during care will be de-identified within the NHS and will be used for care improvement, audit, evaluation, and research. The iCARE system operates a user to data model (with researchers accessing data on NHS Trust Infrastructure). All data access requests are reviewed by the Imperial Data Prioritisation, Access, and Ethic Committee, which provisions ethical access to the de-identified research database based on the overarching NHS Research Ethics Committee database approval. The Access Group, which includes senior clinicians and researchers, members of the Trust's Data Protection Office, the Caldicott Guardian, and lay partners, reviews and recommends data access for projects based on clinical need and patient and public interest.

4. In the background and discussion the commentary could refer more to articles on hospital falls prevention, especially referring to patient and staff involvement in providing data and using data for evidence based practice and workforce redesign & processes. Some examples could include:

Heng, H., (2022). Healthcare professional perspectives on barriers and enablers to falls prevention education: A qualitative study. PLoS ONE, 17(4 April).

Morris, M. E., T (2024). Preventing hospital falls: feasibility of care workforce redesign to optimise patient falls education. Age and Ageing, 53(1), 9 pages.

Shaw, L. K. (2021). Improving hospital falls screening and mitigation using a health professional education framework. Nurse Education Today, 98.

RESPONSE: Thank you for this suggestion. On reflection, we agree that the introduction should elaborate on how data can be harnessed towards falls prevention. As such, we have added the following narrative in the Knowledge-to-practice section (including the citations you have suggested):

Bringing together a learning community with a focus on integrating local data insights with robust evidence could optimise workforce planning and processes towards safe mobility. Research demonstrates that patient and staff education and multi-factorial interventions can reduce hospital falls [2,36,37]; easily accessible local falls data could help to ensure that education programs and falls prevention strategies are tailored to mitigate prevalent falls risks in different inpatient settings.

Reviewer: 2

Dr. Yoona Choi, Seoul National University, Center for Convergence Approaches in Drug Development

Comments to the Author:

Review Checklist Q1, 3, 4.

- The project outline is commendable and demonstrates a clear ambition to contribute meaningfully to the field. However, it could benefit from a more explicit articulation of its specific aims and hypotheses.

RESPONSE: Thank you for this valuable feedback and suggestions on how to enhance the clarity of the paper. This protocol differs from a traditional research study in that the overall aim is to co-produce an intervention rather than answer a specific research question. To address your point, we have added our hypothesis alongside the overall aim, as follows:

Study aim

This study aims to co-produce and formatively evaluate a safe mobility and falls informatics platform in the acute hospital setting to provide automated, real-time insight into inpatient falls. We hypothesise that readily available data on the context, circumstances, mechanisms, and outcomes of falls will enable meaningful quality improvement activity to reduce injurious falls in the inpatient setting.

The insightFall platform

The informatics platform is intended to (1) provide data-driven insight to support the initial learning response when a fall occurs, (2) guide meaningful, coordinated quality improvement activity based on trends in aggregated data, and (3) provide a mechanism for near-real-time monitoring of inpatient falls at trust-level (assurance). Intended users of the insightFall platform are ward managers, matrons, and the Safe Mobility and Falls Prevention specialist clinical team, and Safe Mobility and Falls Prevention Steering Committee.

- The protocol appears overly descriptive in parts, without adequately specifying what will be investigated. Clarifying the research questions and the variables or outcomes of interest would greatly enhance the study's focus and impact.

RESPONSE: Thank you. We believe that the additional clarification we have provided in relation to the aim, hypothesis, and variables of interest (see data elements below), aligned with your feedback, has augmented the manuscript.

- There is a need for more detailed descriptions of the data elements that will be used to investigate these variables or outcomes. For instance, when discussing information extraction, it would be helpful to specify the types of information being sought (e.g., number of falls, timing of falls, events occurring before or after falls).

RESPONSE: Thank you for suggesting that we add more detailed descriptions of the data elements to used to monitor and inform quality improvement activity around inpatient falls. In response to your feedback we have added the following paragraph under the sub-heading 'Data Elements':

Data elements

The data requirements for the insightFall platform be specified by clinical staff during the co-design workshops. Data elements will likely include:

• falls rates (Of note, since raw numbers of falls can be misleading due to variation in bed occupancy and length of stay, rates can be expressed as falls per 1000 occupied bed days [43])

• data from multifactorial risk assessments and care plans (including the risk factors identified, the interventions put in place to mitigate falls risk, and how frequently assessments and care plans were updated by clinical staff [3,40])

• contextual information (e.g. time and location of falls)

• post-falls management (e.g. medical assessment within 30 minutes [39])

• outcomes (e.g. rate of femoral fractures [39]).

The platform will not display metrics on risk categories; Falls Risk Assessment Tools that assign risk scores and place patients in risk categories (e.g. 'no risk, 'low risk', etc.) or are not used in our Trust since robust trials and national guidelines do not support their use [40,44].

- It would be beneficial to outline the expected outcomes more clearly. What insights does the study aim to provide? If the goal is to identify patterns in patient characteristics or situations leading to falls, how will this information be used?

RESPONSE: The goal is to automatically extract data on the context, circumstances, mechanisms, and outcomes of falls; we have articulated this in the new section entitled 'Data elements'. The paragraph immediately after the aim and hypothesis outlines, at a high level, how this information will be used (and by whom):

The informatics platform is intended to (1) provide data-driven insight to support the initial learning response when a fall occurs, (2) guide meaningful, coordinated quality improvement activity based on trends in aggregated data, and (3) provide a mechanism for near-real-time monitoring of inpatient falls at trust-level (assurance). Intended users of the insightFall platform are ward managers, matrons, and the Safe Mobility and Falls Prevention specialist clinical team, and Safe Mobility and Falls Prevention Steering Committee.

- If the study intends to develop a platform based on its findings, how will this platform be utilized? For example, is there an aim to alert medical staff to potential fall risks preemptively? Or just to gather related insights? Clarifying the intended applications of the research findings could significantly enhance the protocol's relevance and applicability.

RESPONSE: We have added a paragraph entitled 'Implementation and evaluation' clarify the intended applications of the insightFall platform and to indicate future implementation and evaluation plans:

The co-produced insightFall platform will be incorporated into Imperial College Healthcare NHS Trust's patient safety incident response plan and policy, in line with NHSE's Patient Safety Incident Response Framework [14]. Reviewing data-driven insights on the informatics platform will be built into the incident response workflow for falls, enabling ward managers and matrons to incorporate data on the context, circumstances, mechanisms and outcomes of falls into ward-level safety improvement activity without the burden of manually collating data from clinical records. At Trust-level, learning from the platform will be built into the cycle of business and work of the Safe Mobility and Falls Prevention Steering Committee and specialist clinical team. Trust, Divisional and Directorate-level data insights will used for quality assurance, and to direct resource towards improvement efforts to priority clinical areas. Reporting a detailed evaluation plan is outside the scope of the current protocol, however, future plans include testing the feasibility of the data-driven quality improvement approach using a mixed-methods stepped-wedged design with rapid-cycle process evaluation to create a regular feedback loop between end-users and the informatics team to enable the platform to be refined during implementation. Trial endpoints will include the uptake and use of the insightFall platform (Qlik Sense audit metrics), quality improvement activity related to safe mobility and falls (Life QI metrics), and rates of injurious falls.

Review Checklist Q 6, 7.

- The abstract mentions exploratory analysis of variety, veracity (accuracy/completeness), and value, yet lacks detailed descriptions of how these metrics are defined and evaluated. Clarification on the methodologies for measuring and analyzing each would provide greater insight into the study's objectives and potential impacts.

RESPONSE: We have extended the paragraph entitles 'Exploratory data analysis' to provide greater clarity on the methodologies of measuring and analysing these properties:

Exploratory data analysis

Based on the co-designed data taxonomies, an historical static dataset (01/11/2022 – 30/04/2023) will be curated comprising de-identified, linked, routine data from electronic patient records (Cerner) and incident reports (Datix) (approximately 800 falls). Since these data are collected as part of routine care delivery and not for the specific purposes of this project, the properties of the data will be explored to determine how they can be transformed into actionable insight [48]. The structure of the data (variety) will be examined to determine whether summary statistics or free-text analysis will be needed. The quality (veracity) of the data will be assessed to determine the proportion of missing, noisy or inconsistent data for each variable [49]. This stage of data pre-processing is important since real world electronic health record data is frequently "messy" due to erroneous or incomplete clinical documentation [49]. Preliminary insights related to the context, circumstances, mechanisms, and outcomes of falls will be extracted and presented alongside the percentage of missing or erroneous values per variable (values outside the expected parameters – e.g. Glasgow Coma Scale of 19). These data will be reviewed by and discussed with the Trust's falls prevention team to understand their potential value for measuring and monitoring the problem of inpatient falls and for informing quality improvement activity.

- Regarding the study population and setting, it's mentioned that de-identified data from electronic patient records will be used. However, specifics on the time frame for data collection, the patient population from which the data will be extracted, etc. are missing. Additionally, an approximate sample size for both patients and participating medical staff should be stated to give a sense of scale and scope.

RESPONSE: We have indicated the timeframe and sample size for the historical falls dataset (see above). We aim to involve approximately 30 members of clinical staff in the co-design –

we have added this detail to the 'Study population and setting' section. We have already indicated, in the PPIEP section, that we are aiming to involve 5 lay partners.

- In the section on prototype usability testing, criteria for what constitutes "usability" are not defined. It would be beneficial to specify what conditions or benchmarks will be used to evaluate usability. Furthermore, recommendations on the appropriate number of staff members to be involved in usability testing would help in understanding the expected reliability and applicability of the testing outcomes.

RESPONSE: We have elaborated on our use of the System Usability Scale as follows:

After the 'think-aloud' part of the interview, interviewees will be asked a series of follow up questions and then invited to complete the System Usability Scale questionnaire [48]. The System Usability Scale is a validated, 10-item questionnaire with Likert-type responses that gives a global view of end-users' subjective assessments of usability (for example: "I thought the system was easy to use"; "I think I would need the support of a technical person to use this system") [48].

The scale has been cited, but the scale could be included as a supplementary file at the Editor's preference.

Reviewer: 3

Dr. Kristiana Ludlow, Macquarie University

Comments to the Author:

This is a well-written manuscript that was clear and engaging. A good overview of previous literature and background context was provided, as well as a strong study rationale. The methods were clearly described; however some details were missing. My feedback to further strengthen the manuscript is below.

1. Can you please explain who "lay partners" might be, e.g., patients, caregivers, members of the public?

RESPONSE: We have clarified in the manuscript that ‘lay partners’ are patient representatives.

2. What activities will the co-design workshops involve? For example, you say “the written content of co-design activities”, but it’s not clear what activities will be conducted beyond verbal discussions.

RESPONSE:

3. Please provide a detailed account of the qualitative data analysis, e.g., analysis steps, who will be involved, software etc.

4. “Content analysis will be used to derive descriptive categories and themes”. Content analysis identifies categories, whereas thematic analysis identifies themes. Will you be using both?

5. How will you move from co-design to usability testing? It seems a step around prototype development is missing.

RESPONSE: Thank you for spotting this important omission. We have added a paragraph entitled ‘Prototype development’, as follows:

Prototype development

The platform prototype will be developed by incorporating the goals and data requirements specified by clinical staff as the end-users and an understanding of the availability and quality of the data. The initial wireframes (high-level platform components) will be presented during the Trust’s annual Falls Summit and feedback will be gathered from around 50 clinical staff (using Mentimeter) to reach consensus on the data elements to be displayed in the insightFall platform. A second round of codesign workshops with end-users will focus on how these data should be displayed. The data will be presented in a variety of ways (tables, visualisations) and end-users will rate the insights based on how easy they are to understand. In the final stage of development, a data developer will create the initial prototupe for user testing in Qlik Sense.

6. How many participants will be involved in the usability testing and co-design workshops?

RESPONSE: We have added participant sample sizes as follows:

In the ‘co-design’ section, we state:

There will be one workshops at each of the three main hospital sites; each workshop will involve up to 15 participants, including clinical staff, patient safety team members, and patient representatives.

In the ‘prototype usability testing’ section, we now state:

We aim to recruit a sample of 20 staff members with diverse professional characteristics (professional role, clinical specialty or directorate, work location) […]

Staff will be asked to ‘think aloud’ whilst simultaneously interacting with the dashboard to elicit their thoughts about its use.

7. Will participants be openly interacting with the dashboard during usability testing or assigned tasks?

RESPONSE: Thank you for questioning this – on reflection, we agree there needed to be more clarity around the think aloud prompts. We have amended the relevant section as follows:

Staff will have the opportunity to openly interact with the dashboard and will receive verbal prompts from the interviewer, for example, ‘What are your first impressions about the platform? What are you thinking now? What makes you think that?’ The prompts will become more task focussed, for example, ‘How might you use the platform to support you with your incident response documentation after a patient has fallen? Talk me through it. How might you use the dashboard to support a quality improvement project around falls in your ward? Show me which data you would look at’.

8. Will participants be given the opportunity to engage in think-aloud before being interviewed? The manuscript currently reads as an interview during a task, rather than think-aloud.

RESPONSE: We have reworded the section outlining the think-aloud approach (as above).

9. An evaluation of the co-design approach will be conducted through web-based questionnaires. What are these questionnaires and how will data be analysed?

RESPONSE: Following the recent publication by Nordin and colleagues in BMJ Open (September 2023) 'Measurement and outcomes of co-production in health and social care: a systematic review of empirical studies', we have refined our approach to evaluating the quality of our co-design, as outlined in the manuscript, as follows:

As recommended by Nordin and colleagues [51], our co-production approach will be evaluated from three perspectives: (1) the outputs of co-production processes, (2) participants' experiences of participating in co-production and the (3) outcomes of co-production processes. The outputs of the co-design workshops and usability testing will consist of three insights reports: the first report will outline the data elements that clinical staff would like to see displayed in the insightFall platform, the second report will outline how clinical staff would like to see data driven insights displayed (including their preferences for different types of visualisations); the final report will bring together System Usability Scale results with the think-aloud findings aligned with the Technology Acceptance Model (perceived usefulness, perceived ease-of-use and intentions to use the platform) [48,50]. Participants' experiences of co-production will be evaluated using a two-item web-based questionnaire administered using Microsoft Forms at the end of each codesign workshop; item one is an overall workshop rating (1 to 5 stars) and item 2 asks participants to justify the rating they gave. The questionnaire was designed to be short and simple based on feedback that completion of the questionnaire should not be burdensome for busy clinical staff who have already given up time to participate in the codesign session. Co-production outcomes will be evaluated using the following metrics: i) the proportion of end-users with Systems Usability Scores >68 in usability testing and ii) the proportion of wards & directorates with sustained adoption at the end of the implementation and evaluation phase.

10. Could you please provide more information about: “Secondary analysis of data collected as part of the trust’s Ward Accreditation Programme and trust-wide administration of the research impact capture tool will also contribute towards the evaluation”.

RESPONSE: These metrics are not included in our revised evaluation approach (see above). This sentence has been removed from the manuscript.

11. Do you have plans to implement and evaluation the platform? While this might be outside the scope of the co-design study, adding some brief information about future plans would be beneficial.

RESPONSE: Thank you – in response to your question/comment, we have added a paragraph entitled ‘Implementation and evaluation’:

The co-produced insightFall platform will be incorporated into Imperial College Healthcare NHS Trust’s patient safety incident response plan and policy, in line with NHSE’s Patient Safety Incident Response Framework [14]. Reviewing data-driven insights on the informatics platform will be built into the incident response workflow for falls, enabling ward managers and matrons to incorporate data on the context, circumstances, mechanisms and outcomes of falls into ward-level safety improvement activity without the burden of manually collating data from clinical records. At Trust-level, learning from the platform will be built into the cycle of business and work of the Safe Mobility and Falls Prevention Steering Committee and specialist clinical team. Trust, Divisional and Directorate-level data insights will used for quality assurance, and to direct resource towards improvement efforts to priority clinical areas. Reporting a detailed evaluation plan is outside the scope of the current protocol, however, future plans include testing the feasibility of the data-driven quality improvement approach using a mixed-methods stepped-wedged design with rapid-cycle process evaluation to create a regular feedback loop between end-users and the informatics team to enable the platform to be refined during implementation. Trial endpoints will include the uptake and use of the insightFall platform (Qlik Sense audit metrics), quality improvement activity related to safe mobility and falls (Life QI metrics), and rates of injurious falls.

12. Please provide a reference(s) for the sentence starting with: “These insights should prompt improvement activity such as the redesign …”

RESPONSE: We have added the appropriate reference – see Hardie (2022).

13. Please provide a reference for the sentence: “Methods to visualise patient safety data can support clinical teams to better understand their safety problems.”

RESPONSE: We have added the appropriate reference – see Ludlow (2021).

VERSION 2 - REVIEW

**Reviewer**	**1**
**Name**	**Morris, Meg**
**Affiliation**	**La Trobe Univ, Room 423 HS3**
**Date**	**13-Apr-2024**
**COI**	**Nil**

The revisions have addressed all of my original questions, thank you.

**Reviewer**	**2**
**Name**	**Choi, Yoona**
**Affiliation**	**Seoul National University**
**Date**	**03-May-2024**
**COI**	**Nothing to declare.**

Most of my previous suggestions, particularly regarding the study's objectives and methods, have been incorporated. This has significantly enhanced the clarity of the study outline and provided sufficient detail in the methods section to allow for the replication of the study.

Just one thing to add: I would recommend that the verification and model refinement of the NLP model be conducted not only qualitatively by the advisory group consisting of clinical experts but also quantitatively, using the commonly accepted evaluation metrics for NLP models such as accuracy, precision, recall, or F-1 score. This should include benchmarks from previous studies to ensure a robust assessment and intuitive interpretation of the results.

**Reviewer**	**3**
**Name**	**Ludlow, Kristiana**
**Affiliation**	**Macquarie University, Australian Institute of Health Innovation**

Date 22-Apr-2024

COI **I have no competing interests to declare**

Thank you for responding to the feedback from myself and the other reviewers. Your changes have strengthened the manuscript and improved the clarity of the of the methods. One of my previous comments was not addressed in the response sheet or the manuscript. Please respond to the following:

Please provide a detailed account of the qualitative data analysis, e.g., analysis steps, who will be involved, software etc.

VERSION 2 - AUTHOR RESPONSE

Responses to reviewers and handling editor

**Reviewer 1 – Comment 1:**<br><br>The revisions have addressed all of my original questions, thank you.	Many thanks for reviewing the revised manuscript. We believe that the amendments made in response to your feedback have substantially improved this paper.
**Reviewer 2 – Comment 1:**<br><br>Most of my previous suggestions, particularly regarding the study's objectives and methods, have been incorporated. This has significantly enhanced the clarity of the study outline and provided sufficient detail in the methods section to allow for the replication of the study.	Thank you once again for your considered feedback and time taken to review our revised manuscript.
**Reviewer 2 – Comment 2:**<br><br>Just one thing to add: I would recommend that the verification and model refinement of the NLP model be conducted not only qualitatively by the advisory group consisting of clinical experts but also quantitatively, using the commonly accepted evaluation metrics for NLP models such as accuracy, precision, recall, or F-1 score. This should include benchmarks from previous studies to ensure a robust assessment and intuitive interpretation of the results.	Thank you for your helpful comment. We agree with your suggestions and have added further detail into the manuscript about our planned quantitative appraisal of the NLP model, using the F1 score:<br><br>*"As a final step, the NLP models will be applied to the most recent one year of unseen falls documentation. The advisory group will manually tag the concept classes without viewing the predicted classes to avoid bias, and inter-rater agreement will be calculated at the note level to appraise the level of agreement. To evaluate the overall performance of the NLP models, the F1 score will be calculated for each label. Previous work has shown that rule-based models applied to falls documentation can achieve an F1 score of at least 70% [47,48]. Given the specific requirements of the clinical application, this will be set as the target before the model is deployed for real-time use."* (**Page 12**)

**Reviewer 3 – Comment 1:**<br><br>Thank you for responding to the feedback from myself and the other reviewers. Your changes have strengthened the manuscript and improved the clarity of the of the methods.	Thank you for your helpful feedback. We very much appreciate the time taken to review our work and agree that revisions made in line with reviewer comments have strengthened the manuscript.
**Reviewer 3 – Comment 2:**<br><br>One of my previous comments was not addressed in the response sheet or the manuscript. Please respond to the following:<br><br>Please provide a detailed account of the qualitative data analysis, e.g., analysis steps, who will be involved, software etc.	Thank you for bringing this to our attention. In response to your comment, we have introduced a more detailed explanation of the planned qualitative data analysis as follows:<br><br>*"Qualitative data from the audio-recorded think aloud interviews will be transcribed verbatim and analysed within NVivo software using Framework Analysis [52], drawing on the Technology Acceptance Model as a preliminary framework [53]. Two researchers (RLe, PA) will carry out the Framework Analysis according to the five-step analytical approach described by Ritchie and Spencer [52], which permits systematic and comprehensive analysis of qualitative data generated within applied research. The researchers will begin with a process of familiarising themselves with transcribed data, making notes as to recurrent ideas and concepts to shape the identification of an initial thematic framework. Addressing our aim to understand the usability of the informatics platform, the initial thematic framework will be informed by concepts derived a priori from the Technology Acceptance Model [53], including 'perceived usefulness', 'perceived ease of use', and 'behavioural intention to use', together with additional themes arising from study data. The thematic framework will be systematically applied to all transcripts, with data abstracted, summarised, and synthesised into a framework matrix in a process termed 'charting'. A final interpretation stage will encompass characterisation of the range and nature of participant perspectives on system usability, with identification of ways in which prototype usability could be optimised to enhance user experience and intervention uptake."* **(Pages 14-15)**
